# An ethnobotanical study of medicinal plants used by the Tengger tribe in Ngadisari village, Indonesia

**DOI:** 10.1371/journal.pone.0235886

**Published:** 2020-07-13

**Authors:** Nurul Jadid, Erwin Kurniawan, Chusnul Eka Safitri Himayani, Andriyani, Indah Prasetyowati, Kristanti Indah Purwani, Wirdhatul Muslihatin, Dewi Hidayati, Indah Trisnawati Dwi Tjahjaningrum

**Affiliations:** Department of Biology, Institut Teknologi Sepuluh Nopember, Surabaya, Indonesia; University of Poonch Rawalakot, PAKISTAN

## Abstract

The people of Tengger, Indonesia have used plants as traditional medicine for a long time. However, this local knowledge has not been well documented until recently. Our study aims to understand the utilization of plants in traditional medicine by the people of Tengger, who inhabit the Ngadisari village, Sukapura District, Probolinggo Regency, Indonesia. We conducted semi-structured and structured interviews with a total of 52 informants that represented 10% of the total family units in the village. The parameters observed in this study include species use value (SUV), family use value (FUV), plant part use (PPU), and the relative frequency of citation that was calculated based on fidelity level (FL). We successfully identified 30 species belonging to 28 genera and 20 families that have been used as a traditional medicine to treat 20 diseases. We clustered all the diseases into seven distinct categories. Among the recorded plant families, Poaceae and Zingiberaceae were the most abundant. Plant species within those families were used to treat internal medical diseases, respiratory-nose, ear, oral/dental, and throat problems. The plant species with the highest SUV was *Foeniculum vulgare* Mill. (1.01), whereas the Aloaceae family (0.86) had the highest FUV. *Acorus calamus* L. (80%) had the highest FL percentage. The leaves were identified as the most used plant part and decoction was the dominant mode of a medicinal preparation. Out of the plants and their uses documented in our study, 26.7% of the medicinal plants and 71.8% of the uses were novel. In conclusion, the diversity of medicinal plant uses in the Ngadisari village could contribute to the development of new plant-based drugs and improve the collective revenue of the local society.

## Introduction

The interaction between humans and plants has been long described as one of the factors influencing human civilization, especially in medicinal fields [1]. Documentation of the medicinal use of plants through ethnobotanical studies enables the development of contemporary drugs and treatments as well as for plant conservation [2, 3]. Many ethnobotanical studies around the world, including in Indonesia, report the use of herbal plants for the healing process, which has been in use for several generations in their respective societies [4, 5]. Though the cultural diversity in Indonesia contributes to the extensive this traditional knowledge [6], access to this is limited. Traditional knowledge is usually passed on orally and often person-specific [7]. Therefore, the knowledge is often owned by tribal leaders, village heads, elders, heads of *kampung* (small village), or traditional healers in the particular community or tribe [8].

Indonesia has around 40,000 different plant species, of which approximately 6,000 are used for traditional healing processes [4], especially in certain tribal areas including Bromo Tengger Semeru National Park (BTSNP) [9]. BTSNP is designated as a national park because of its fascinating vegetation (about 600 floral species) and is home to the unique Tengger tribe. Some plants have been cultivated for daily consumption and trading whereas others are naturally found and used for particular purposes such as tribal ceremonies and medicinal uses [9]. People of Tengger are distributed in buffer zone villages around the BTSNP including Ngadisari village [10].

The Tengger people use plants from the BTSNP for traditional ceremonies [11], as well as medicinal applications [12], industrial materials, food sources, and building materials in some buffer village areas [13]. However, there are no reports regarding the ethnobotanical aspect of medicinal plants used in these buffer village areas in the BTSNP by the Tengger people. The present study documents the medicinal plant species and traditional knowledge of the Tengger tribe who inhabit the Ngadisari village in the BTSNP, Indonesia.

## Materials and methods

### Study area

**Fig 1:**
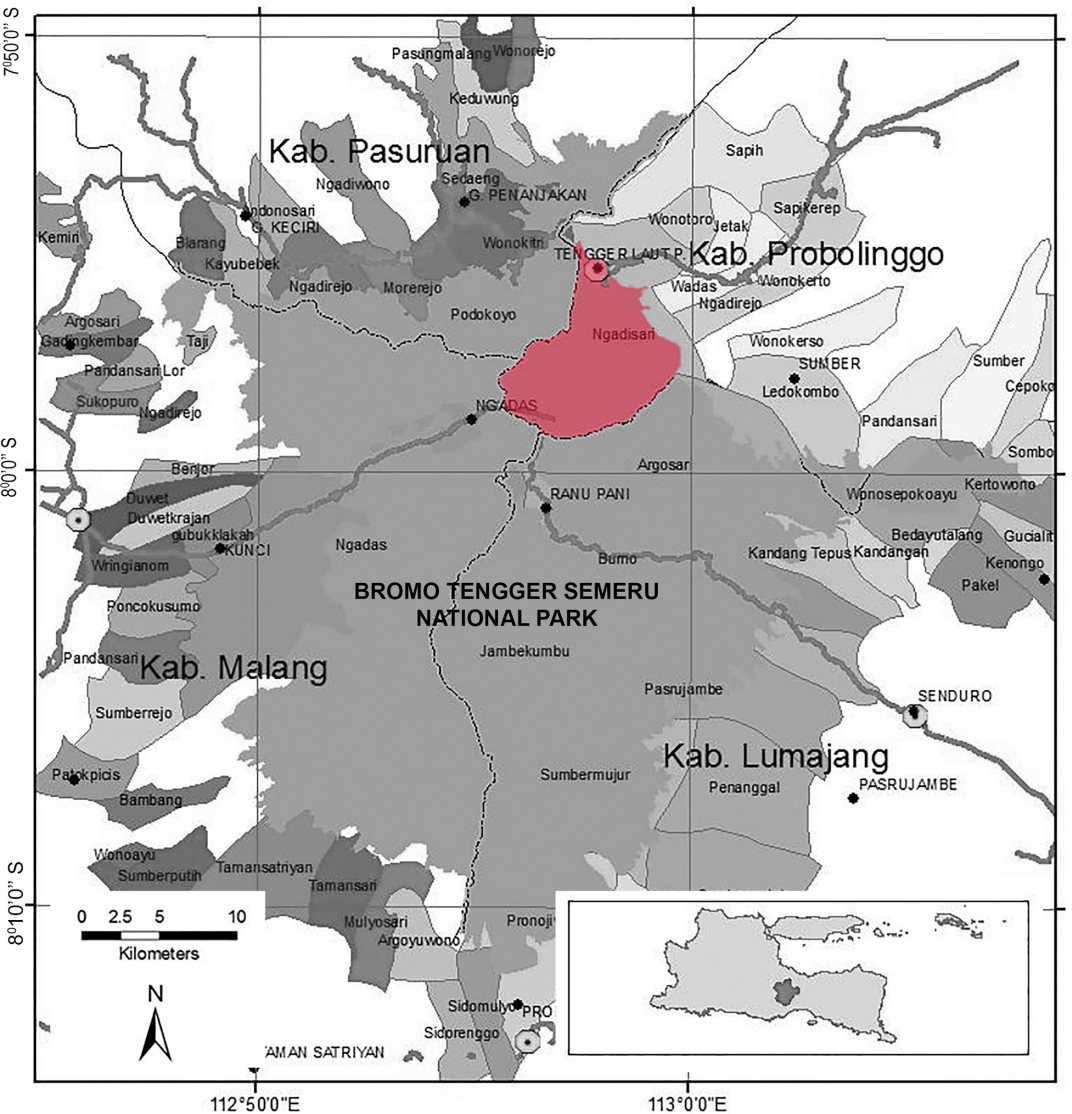
Location of the study area. Ngadisari village (A, red color) is located within the Bromo Tengger Semeru National Park Indonesia. This figure is similar but not identical to the original image obtained from [15] under a CC BY license and is used for illustrative purposes only.

This study was carried out in Ngadisari village, which belongs administratively to the Sukapura district in Probolinggo Region of the Republic of Indonesia. It is located at 7° 55’ 18” S 112° 57’ 21” E around the Bromo Tengger Semeru National Park (BTSNP) (Fig 1). Ngadisari village is situated at an altitude of 1800–1950 meters above sea level. The total area of the study comprised 4,993 km^2^. Like other regions in Indonesia, Ngadisari village has only two seasons; dry and rainy. The rainy season spans the months of November-May, whereas the dry season spans June-October. The present study was conducted from 2018–2019. Most inhabitants belonged to the Tengger tribe and rely on agriculture. According to the Indonesian Statistics Bureau (BPS) data, the Ngadisari village has a population of 1,543 inhabitants or about 507 family units (households) [14]. The population consists of 742 males and 801 females. The Welsh onion (*Allium fistulosum* L.), potato (*Solanum tuberosum* L.), cabbage (*Brassica oleracea* L.), carrot (*Daucus carota* L.) and corn (*Zea mays* L.) are examples of plants that contribute to the income of these communities [9].

### Data collection

This study was authorized (SK No. 091650/IT2.VII/HK.00.02/2018) by the Institute of Research and Community Service (LPPM) of the Institut Teknologi Sepuluh Nopember (ITS), Surabaya, Indonesia. Verbal informed consent was obtained from each informant before conducting the interview process.

Data collection was obtained through semi-structured and structured interviews with informants who knew or used plants as medicine. This technique is commonly used in ethnobotanical studies [16]. Interviews were conducted with selected informants including about 10% of the total heads of family units (52 informants) to determine and explore the traditional knowledge regarding the utilization of medicinal plant species, their usefulness, the utilized part, mode of preparation, or method of processing the plants. All of the head family units were males since norms, values, and local wisdom are based on patriarchal culture [17]. The age of the informants ranged from 25 to more than 45 years, where four were between the ages of 25–30, sixteen were ranging from 31–35, eighteen were between 36–40, nine were ranging from 41–45, and five informants were older than 45 years. The interview activities were carried out in their entirety using a questionnaire. Informant selection was based on the Snowball Sampling technique, by determining the key person. A key-person is one who possesses strong power within society. The subsequent informants are determined by the direction of the previous respondents.

### Taxonomical identification and herbarium

Taxonomical identification was conducted to verify the samples that were raised during the interviews. An herbarium was also prepared to obtain dry specimens supporting the taxonomical identification. However, the herbarium method was used only for unknown species. Photo documentation and herbarium of medicinal plants were then identified by Christin Risbandini under the laboratory of plant bioscience and biotechnology, Institut Teknologi Sepuluh Nopember, Indonesia using key dichotomy and some references [18, 19].

### Disease classification and grouping

Diseases that commonly occur in the Indonesian region were grouped into seven categories including gastrointestinal disorders (GI) (diarrhea, nausea, vomiting, stomach ache, gastric problems, loss of appetite, colic, flatulence, dysentery); dermatological diseases (DO) (skin burns, skin spots, skin rashes, boils, cut, wounds, hair problems, ectoparasites); urogenital and gynecological problems (UGP) (sexual problems including frigidity, lack of libido, infertility, gonorrhea, diuretic, aphrodisiac, menstrual disorders); skeletomuscular disorders (SD); internal medical diseases (IM) (diabetes, cancers, and tumors, hypertension, piles/hemorrhoids); respiratory-nose, ear, oral/dental, throat problems (RT) (asthma, nose bleeding, sinusitis, earache, throat shore, dental problems); and others (OT) (motion sickness).

### Data analysis

#### Fidelity Level (FL)

The relative frequency of citation was calculated using the fidelity level (FL) formula according to Friedman *et al*. [20] and Ouedraogo *et al*. [21]. FL is the percentage of informants who claim to use certain plant species for particular healing processes. This reflects the preference of people for a specific plant species in a particular medicinal treatment. It was calculated using the following equation:

$$FL\;(\%)=\frac{Np}{N}x\;100$$ (1)

Where *Np* is the number of informants who mentioned or claimed the use of plant species for a particular healing process/medicinal treatment. *N* is the total number of informants who cited the plant species for various kinds of medicinal treatment.

#### Species Use Value (SUV)

SUV signifies the value of a medicinal plant species used by the people from Ngadisari village. It is calculated as the sum of the informant species use values (*UV*_*is*_) for a particular medicinal species divided by the total number of informants (*N*_*i*_). The SUV was calculated according to Hoffman and Gallaher [22] as follows:

$$SUV=\frac{\sum UVis}{{(n}_{i})}$$ (2)

#### Family Use Value (FUV)

FUV was calculated as described by Phillips and Gentry [23], signifying the use value of a given plant family that is used as medicine by the people from Ngadisari village. The calculation follows the below equation:

$$FUV=\frac{\sum UVs}{{(n}_{s})}$$ (3)

Where ∑*UV*_*s*_ represents the sum of the use values for all species belonging to a particular family divided by the total number of species in the same family.

#### Plant Part Value (PPV)

The plant part value is presented as the percentage of utilized parts of plants (stem, leaves, root, fruit, bark, and flower) that are used as medicinal bioresources. The PPV is calculated according to Gomez-Beloz [24] as follows:

$$PPV(\%)=\frac{\sum {RU}_{\left(plant\;part\right)}}{\sum RU}x\;100$$ (4)

Where ∑*RU*_*(plant part)*_ and ∑*RU* represent the sum of the cited plant parts and the total number of cited uses for a given plant, respectively.

## Results and discussion

### Utilization of plant species as traditional medicine by Tengger tribe in Ngadisari village

The people of Tengger receive their knowledge of traditional medicine from their ancestors. This knowledge is inherited and subsequently preserved across generations [12]. We found 30 plant species that are used in traditional medicine. Among them, eight plants (26.7%) were recorded for the first time, compared with the previous study [9, 11, 12]. They were *Mandevilla sanderi* (Hemsl.) Woodson, *Jatropha curcas* L., *Cymbopogon nardus* (L.) Rendle), *Microsorum buergerianum* (Miq.) Ching., *Paederia foetida* L., *Solanum muricatum* Ait., *Zingiber zerumbet* (L.) Sm., and *Senna alata* (L.) Roxb. Also, different medicinal uses for known plants (71.8%) were observed in the present study, compared to the study conducted by Batoro [9] (Table 1).

**Table 1:** Disease categories, health-related problems, and medicinal plants used in Ngadisari village.

No	Disease Categories	Specified disease name	Plant family	Plant species	Common name	Local name	Plant part used	Mode of preparation
1	Internal medical diseases	Hypertension	Apiaceace	*Apium graveolens* L.	Celery	*Seledri*	Leaves	Eaten raw, decoction
			Solanaceae	*Physalis angulata* L.	Cutleaf ground cherry	*Keciplukan*	Leaves	Decoction
			Solanaceae	*Solanum muricatum* Ait.	Pepino dulce, sweet cucumber	*Buah Melodi*	Fruit	Eaten raw
			Zingiberaceae	*Zingiber zerumbet* (L.) Sm.	Bitter ginger	*Lempuyang*	Rhizome	Eaten raw
		Fever	Acoraceae	*Acorus calamus* L.	Sweet flag, calamus	*Dringu*	Leaves	Pounded
			Liliaceae	*Allium cepa* L.	Onion	*Bawang merah tengger*	Bulb	Burned
			Poaceae	*Cymbopogon nardus* (L.) Rendle	Citronella grass	*Serai*	Leaves	Squeezed
			Poaceae	*Saccharum officinarum* L.	Sugarcane	*Tebu merah*	Stem	Burned
			Zingiberaceae	*Curcuma domestica* Val.	Curcuma	*Kunyit*	Rhizome	Shredded
		Nose bleeding	Asteraceae	*Artemisia vulgaris* L.	common wormwood	*Ganjan*	Leaves	Rolled up
		Hemorrhoid	Myrtaceae	*Psidium guajava* L.	Guava	*Jambu klutuk*	Leaves	Pounded
			Solanaceae	*Physalis angulata* L.	Cutleaf Ground Cherry	*Keciplukan*	Leaves	Decoction, pounded
			Clusiaceae	*Garcinia mangostana* L.	Mangosteen	*Manggis*	Stem bark	Burned
2	Urogenital and gynecological problems	Leucorrhea	Piperaceae	*Piper betle* L.	Betelvine	*Sirih*	Leaves	Decoction
			Rubiaceae	*Paederia foetida* L.	Stinkvine	*Kesimbukan*	Leaves	Decoction
3	Dermatological diseases	Hair problems	Arecaceae	*Cocos nucifera* L.	Coconut	*Kelapa*	Fruit	Decoction
			Aloaceae	*Aloe vera* (L.) Burm. f.	Barbados aloe	*Lidah Buaya*	Leaves	Smeared
		Urticaria/hives	Apiaceace	*Foeniculum vulgare* Mill.	Fennel	*Adas*	Leaves	Decoction, Pounded
			Piperaceae	*Piper betle* L.	Betelvine	*Sirih*	Leaves	Decoction
			Polypodiaceae	*Microsorum buergerianum* (Miq.) Ching.	Microsorum	*Pangotan*, *paduka aji*	Leaves	Decoction
		Ringworm	Fabaceae	*Senna alata* (L.) Roxb.	Candle bush	*Ketepeng*	Leaves	Pounded, decoction
		Skin burn	Aloaceae	*Aloe vera* (L.) Burm. f.	Barbados aloe	*Lidah buaya*	Leaves	Smeared
4	Respiratory-nose, ear, oral/dental, throat problems	Cough	Liliaceae	*Allium fistulosum* L.	Welsh onion	*Bawang prei*	Leaves	Burned
			Apiaceace	*Foeniculum vulgare* Mill.	Fennel	*Adas*	Leaves	Decoction
			Rutaceae	*Citrus aurantium* L.	Lime	*Jeruk Nipis*	Fruit	Squeezed
			Zingiberaceae	*Zingiber officinale* Rosc.	Ginger	*Jahe*	Rhizome	Pounded, decoction
			Zingiberaceae	*Kaempferia galanga* L.	Chinese ginger, aromatic ginger	*Kencur*	Rhizome	Burned
		Mouth ulcer, sprue	Euphorbiaceae	*Jatropha curcas* L.	Jatropha	*Jarak Pagar*	Stem	Smeared
		Asthma	Poaceae	*Cymbopogon nardus* (L.) Rendle	Citronella grass	*Serai*	Leaves	Decoction
		Heatiness	Poaceae	*Imperata cylindrica* (L.) P. Beauv.	Cogon grass	*Alang-alang*	Leaves	Decoction
		Eye Irritation	Apocynaceae	*Mandevilla sanderi* (Hemsl.) Woodson	Brazilian jasmine	*Bunga Terompet*	Gum	Dropped
5	Skeleto-muscular disorders	hyperuricemia	Euphorbiaceae	*Jatropha curcas* L.	Jatropha	*Jarak Pagar*	Leaves	Decoction
		Muscle soreness	Poaceae	*Dendrocalamus asper* (Schult. f.) Backer ex Heyne	Dragon bamboo, giant bamboo	*Bambu betung*	Stem	Pounded
6	Gastrointestinal disorders	Diarrhea	Apiaceace	*Coriandrum sativum* L.	Coriander	*ketumbar*	Stem	Burned
			Convolvulaceae	*Ipomoea paniculata* Burm. f.	Bindweed	*Tirem*	Leaves	Decoction
			Myrtaceae	*Psidium guajava* L.	Guava	*Jambu klutuk*	Fruit	Eaten raw
		Constipation	Brassicaceae	*Brassica* sp.	Mustard	*Sawi Tengger*	Leaves	Decoction
		Worm disease	Piperaceae	*Piper betle* L.	Betelvine	*Sirih*	Leaves	Decoction
7	Others	Motion sickness	Apiaceace	*Foeniculum vulgare* Mill.	Fennel	*Adas*	Leaves	Squeezed

The highest number of species in one category was found in the category of IM with 12 species, followed by nine species in RT and six species in DD (Table 1). We also observed that five species found in this study were used to treat more than one disease in distinct categories. For instance, fennel, locally named *Adas* (*Foeniculum vulgare* Mill.) has been used to treat urticaria/hives (DD), cough (RT), and to overcome motion sickness (OT). Betelvine (*Piper betle* L.) has also been used for more than one disease including leucorrhoea (UGP), hives or urticaria (DD), and worm disease (GI). This demonstrated that the use value of these species is quite high compared with that of other medicinal plants [22].

### Species and family use value

**Fig 2:**
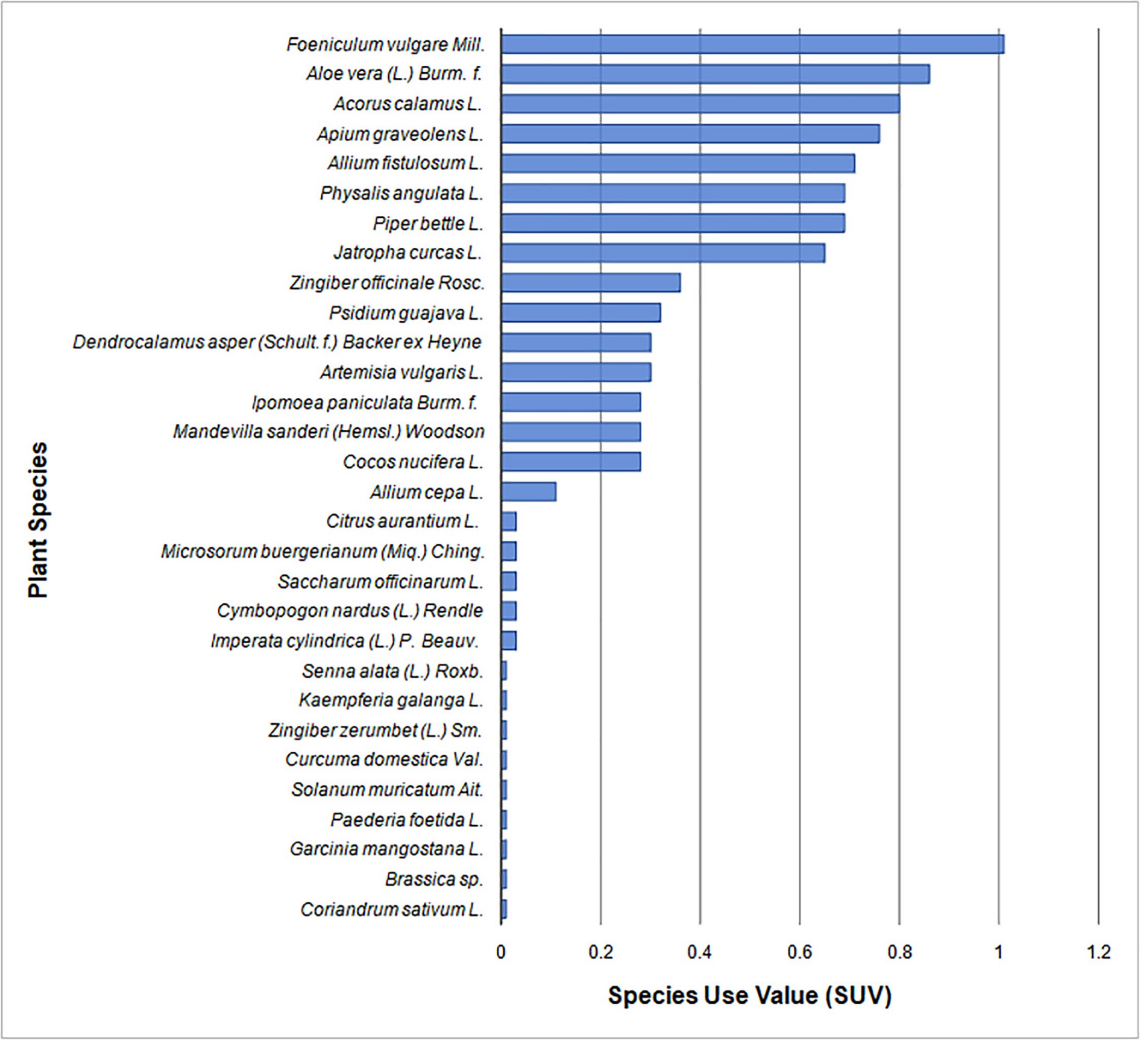
Species Use Value (SUV) of medicinal plants found in the Ngadisari village, Indonesia.

Species use value demonstrates the value of a medicinal plant species used by the people from Ngadisari village. Our results revealed that the SUV of the reported plants varied from 0.01 to 1.01 (Fig 2). Five species showed the highest SUV: *Foeniculum vulgare* Mill. (1.01), *Aloe vera* (L.) Burm. f. (0.86), *Acorus calamus* L. (0.8), *Apium graveolens* L. (0.76), and *Allium fistulosum* L. (0.71). Previous studies also demonstrated that fennel is frequently used as medicinal plants in Indonesia [12, 25] and is abundantly present in this region [9]. Our data showed that *F*. *vulgare* is categorized as a plant used to treat dermatological problems (DO). People of Tengger inhabiting Ngadisari village use *F*. *vulgare* to treat urticaria, hives, or itching. Our results are also in accordance with other studies that revealed *F*. *vulgare* as a traditional medicine for people suffering from itching or other dermatitis problems [26].

Similar medicinal uses of *Aloe vera* (L.) Burm. f., *Acorus calamus* L., *Apium graveolens* L., and *Allium fistulosum* L. have been reported in previous ethnobotanical studies. For example, Reimers *et al*. [27] and Salehi *et al*. [28] reported the use of *Aloe vera* to treat hair problems. Meanwhile, the use of *Acorus calamus* L. to treat fever has been reported by Rajput *et al*. [29]. Also, *A*. *graveolens* and *A*. *fistulosum* L. have been used by traditional Chinese and Indonesian people to reduce blood pressure and cough, respectively [30, 31]. Finally, nine species were reported to have low SUV (0.01) in the present study (Fig 2). High or low SUV may be due to extensive or minimum ethnobotanical uses of the reported species, respectively. Similar results were also reported by Hussain *et al*. [32], where the highest SUV represents the most exploited medicinal plants used to treat a specific ailment.

In total, 30 medicinal plant species have been recorded in our study. All belong to 20 different families, with Poaceae and Zingiberaceae being dominant in the study area (each consisting of four species) followed by Apiaceae (three species). The remaining families were represented by one or two species (Table 2). Poaceae and Zingiberaceae were the most representative medicinal plant families in our study. This finding might be due to the high accessibility of these species in that region. This further supports that dominant plant families and species are commonly used by local people for disease treatment [32]. Moreover, most of the species within both families are cultivated by people of Tengger in the Ngadisari village. The occurrence of dominant plant species and families in the study area is also related to favorable climate and environmental conditions [33, 34]. As a result of the abundance, these species are commonly used as a basic ingredient of *Jamu—*an Indonesian traditional medicine [35]. Sharifi-Rad *et al*. [36] also described that plants from the Zingiberaceae family are a potential source of bioactive phytochemical.

**Table 2:** Family Use Value (FUV) of medicinal plants found in Ngadisari village, Indonesia.

No.	Plant Family	FUV	Number of Species	Local name (Plant species)/voucher number
1	Apiaceace	0.59	3	Adas (*Foeniculum vulgare* Mill.) / NAD-003
				Sledri (*Apium graveolens* L.) / NAD-005
				Tumbar (*Coriandrum sativum* L.) / NAD-001
2	Acoraceae	0.8	1	Dringu (*Acorus calamus* L.) / NAD-030
3	Arecaceae	0.28	1	Kelapa (*Cocos nucifera* L.) / NAD-019
4	Asteraceae	0.30	1	Ganjan (*Artemisia vulgaris* L.) / NAD-021
5	Aloaceae	0.86	1	Lidah buaya (*Aloe vera* (L.) Burm. f.) / NAD-006
6	Apocynaceae	0.28	1	Bunga trompet (*Mandevilla sanderi* (Hemsl.) Woodson) / NAD-029
7	Brassicaceae	0.01	1	Sawi tengger (*Brassica* sp.) / NAD-002
8	Clusiaceae	0.01	1	Manggis (*Garcinia mangostana* L.) / NAD-023
9	Euphorbiaceae	0.65	1	Jarak Pagar (*Jatropha curcas* L.) / NAD-015
10	Liliaceae	0.41	2	Bawang prei (*Allium fistulosum* L.) / NAD-010
				Bawang merah Tengger (*Allium cepa* L.) / NAD-009
11	Myrtaceae	0.32	1	Jambu (*Psidium guajava* L.) / NAD-014
12	Piperaceae	0.69	1	Sirih (*Piper betle* L.) / NAD-008
13	Poaceae	0.1	4	Serai (*Cymbopogon nardus* (L.) Rendle) / NAD-017
				Bambu betung (*Dendrocalamus asper* (Schult. f.) Backer ex Heyne) / NAD-022
				Tebu merah (*Saccharum officinarum* L.) / NAD-018
				Alang-alang (*Imperata cylindrica* (L.) P. Beauv.) / NAD-016
14	Polypodiaceae	0.03	1	Pangotan (*Microsorum buergerianum* (Miq.) Ching.) / NAD-012
15	Rutaceae	0.03	1	Jeruk nipis (*Citrus aurantium* L.) / NAD-013
16	Rubiaceae	0.01	1	Kesimbukan (*Paederia foetida* L.) / NAD-024
17	Solanaceae	0.35	2	Keciplukan (*Physalis angulata* L.) /NAD-011
				Buah melody (*Solanum muricatum* Ait.) / NAD-025
18	Convolvulaceae	0.28	1	Tirem (*Ipomoea paniculata* Burm. f.) / NAD-020
19	Zingiberaceae	0.10	4	Jahe (*Zingiber officinale* Rosc.) / NAD-004
				Kunyit (*Curcuma domestica* Val.) / NAD-028
				Lempuyang (*Zingiber zerumbet* (L.) Sm.) / NAD-007
				Kencur (*Kaempferia galanga* L.) / NAD-026
20	Fabaceae	0.01	1	Ketepeng (*Senna alata* (L.) Roxb.) / NAD-027

The total number of species within a given family has been calculated to obtain their FUV. Our results showed that Aloaceae had a high FUV (0.86), followed by Acoraceae (0.80), Piperaceae (0.69), and Euphorbiaceae (0.65). Other families represented low FUV (< 0.60) (Table 2). High values of FUV might be because the plant species were cited by a large number of people in the study area. In addition, some reports have described similar results. For example, *A*. *vera* or locally named as crocodile’s tongues has been frequently used in some regions such as Southern Africa [37], Asia [38], Nigeria [39], and India [40] to treat dry skin, for improving skin integrity, and to decrease the appearance of acne, skin burn, and wrinkles.

*A*. *calamus* is also cited by other ethnobotanical studies around the world including China [41], India [42], Nepal [43] to treat fever, diarrhea, bronchitis, tumors, skin diseases, and cough treatment [29]. Reported species from Piperaceae has also highly cited in the previous study [11]. Finally, another high FUV in this study was obtained by Euphorbiaceae with only one species (*Jatropha curcas*). The Tengger people use this Barbados nut species to treat mouth ulcers and hyperuricemia. Our data supports other studies; for example, Abdelgadir and Staden [44] reported that its latex is used for ailments such as headache, toothache, mouth ulcers, cold, and cough. Abu Bakar *et al*. [45] also reported that *J*. *curcas* is potentially used to treat hyperuricemia.

### Fidelity level

According to Imran *et al*. [46], the fidelity level (FL) is useful to determine the level of species importance in relation to a particular disease. FL shows the percentage of respondents who mention the use of a plant species for the same main purpose. Ouedraogo *et al*. [21] reported that relative frequency of citation could also be counted based on FL. This is designed to measure species importance for specific purposes. Our results showed that FL of the 30 plant species ranged from 1.92 to 80% (Table 3). *A*. *calamus* demonstrated the highest FL for fever (80%), followed by *A*. *graveolens* (76.92%) and *A*. *fistulosum* (71.15) for treating hypertension and cough, respectively. Based on a previous study, plants with a high percentage of FL are more frequently used as bio-pharmacological resources [47] and should be considered for further conservation program [48] bioassays and phytopharmacological investigation [49, 50].

**Table 3:** Fidelity Level (FL) of medicinal plants in Ngadisari village, Indonesia.

Disease categories	Plant Species	Specified disease name	Fidelity level (%)
Internal medical diseases	*Apium graveolens* L.	Hypertension	76.92
	*Physalis angulata* L.	Hypertension	38.46
	*Solanum muricatum* Ait.	Hypertension	1.92
	*Zingiber zerumbet* (L.) Sm.	Hypertension	1.92
	*Acorus calamus* L.	Fever	80
	*Allium cepa* L.	Fever	11.50
	*Cymbopogon nardus* (L.) Rendle	Fever	1.92
	*Saccharum officinarum* L.	Fever	3.84
	*Curcuma domestica* Val.	Fever	1.92
	*Artemisia vulgaris* L.	Nose bleeding	30.76
	*Psidium guajava* L.	Hemorrhoid	1.92
	*Physalis angulata* L.	Hemorrhoid	30.76
	*Garcinia mangostana* L.	Hemorrhoid	1.92
Ureno-genital and gynaecological problems	*Piper bettle* L.	Leucorrhoea	42.30
	*Paederia foetida* L.	Leucorrhoea	1.92
Dermatological diseases	*Cocos nucifera* L.	Hair problems, hair nourisment	28.84
	*Aloe vera* (L.) Burm. f.	Hair problems, hair nourisment	65.38
	*Foeniculum vulgare* Mill.	Itchy, urticaria/hives	36.53
	*Piper bettle* L.	Itchy, urticaria/hives	3.38
	*Microsorum buergerianum* (Miq.) Ching.	Itchy, urticaria/hives	3.84
	*Senna alata* (L.) Roxb.	Ringworm	1.92
	*Aloe vera* (L.) Burm. f.	Skin burn	21.15
Respiratory-nose, ear, oral/dental, throat problems	*Allium fistulosum* L.	Cough	71.15
	*Foeniculum vulgare* Mill.	Cough	42.30
	*Citrus aurantium* L.	Cough	3.84
	*Zingiber officinale* Rosc.	Cough	36.54
	*Kaempferia galanga* L.	Cough	1.92
	*Jatropha curcas* L.	Sprue, mouth ulcer	65.38
	*Cymbopogon nardus* (L.) Rendle	Asthma	1.92
	*Imperata cylindrica* (L.) P. Beauv.	Heatiness	3.84
	*Mandevilla sanderi* (Hemsl.) Woodson	Eye irritation	28.84
Skeleto-muscular disorders	*Jatropha curcas* L.	Hyperuricemia	3.38
	*Dendrocalamus asper* (Schult. f.) Backer ex Heyne	Muscle soreness	30.76
Gastro-intestinal disorders	*Coriandrum sativum* L.	Diarrhea	1.92
	*Ipomoea paniculata* Burm. f.	Diarrhea	28.84
	*Psidium guajava* L.	Diarrhea	32.69
	*Brassica* sp.	Constipation	1.92
	*Piper bettle* L.	Worm disease	23.07
Others	*Foeniculum vulgare* Mill.	Motion sickness	3.83

Some species have low percentage of FL (1.92%) related to various diseases (Table 3). Examples include, *S*. *muricatum*, *Z*. *zerumbet*, *C*. *nardus*, *C*. *domestica*, *P*. *guajava*, *G*. *mangostana*, *P*. *foetida*, *S*. *alata*, *K*. *galanga*, *C*. *sativum*, *Brassica* sp. Low fidelity levels might also explain the low abundance of plant species in this region. Furthermore, it might also indicate that there is little information about the use of this medicinal plant among the people of Tengger in the Ngadisari village. Even though some plants possess low FL, these species should not be abandoned to preserve traditional knowledge of the society in treating some diseases as reported by Chaachouay *et al*. [51].

### Plant part use and mode of preparation

**Fig 3:**
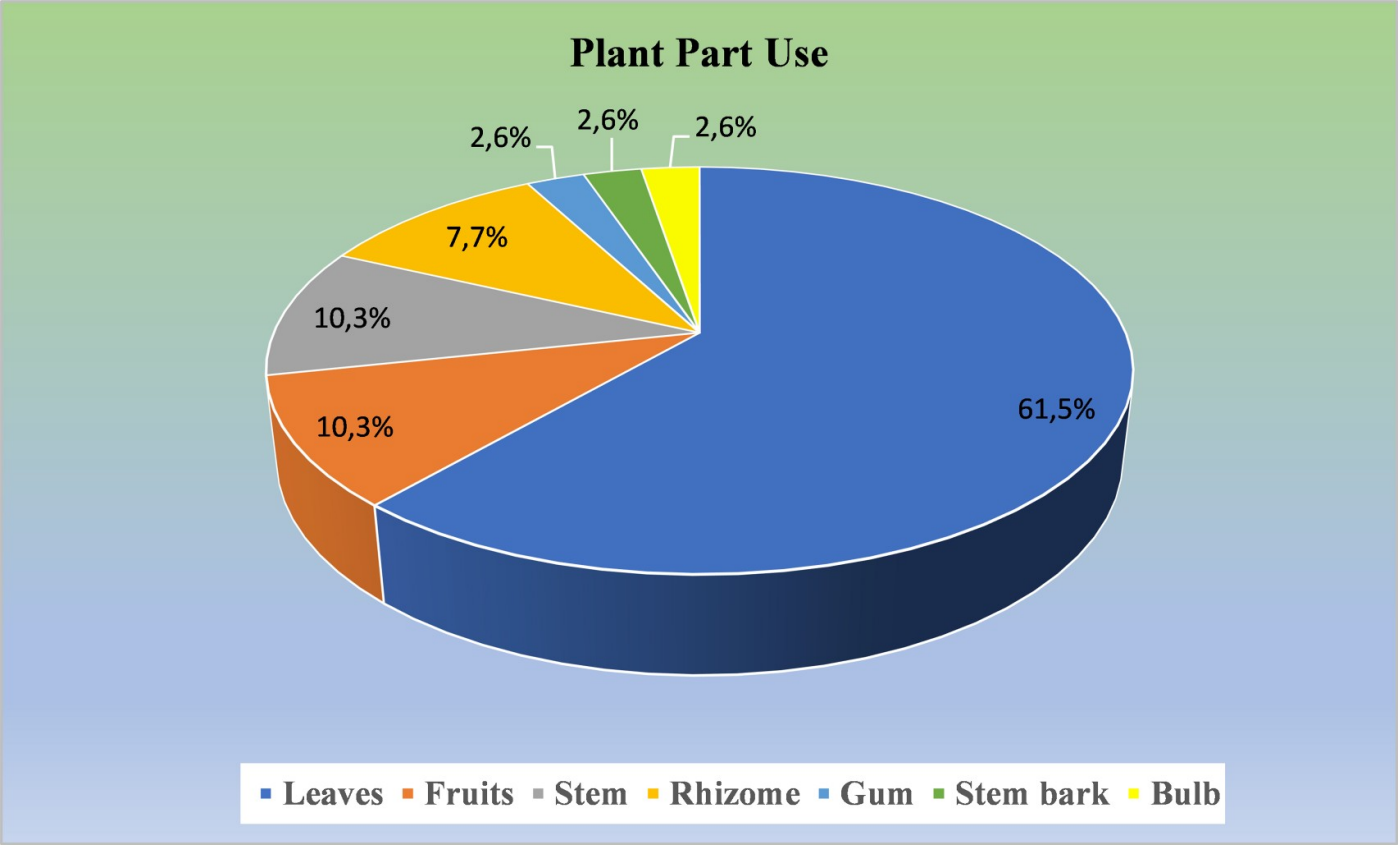
Percentage of medicinal plant part use for herbal preparation in Ngadisari village, Indonesia.

According to Hoffman and Gallaher [22], calculating the use of plant parts (Plant Part Use) is useful to determine the dominant plant parts being used as medicinal ingredients. Plant parts are capable of accumulating diverse and interesting natural compounds. They attract attention because of their ability to act as factories, producing and offering important pharmaceutical potential [52]. Our results showed that leaves were the most predominantly utilized plant parts at 61.5%, while gum, stem bark and bulb represent parts that are infrequently used by people of Ngadisari (Fig 3).

Leaves are the major plant components commonly reported to be used as herbal medicine materials in Indonesia [8, 53] and also in other countries [54–56]. Leaves are common and favorite parts used for medicinal treatment preparation because of easy handling and sustainability [57, 58]. The latter is linked to the survival rate of medicinal plants. Removing the leaves biomass within reasonable limits does not interfere with the plant life, compared to collecting the stem, root, or whole plant, which may risk the plant life [59]. Moreover, many reports have showed that leaves contain diverse plant secondary metabolites [60]. In the present study, no data have been obtained for the use of flowers as medicinal materials. This might offer other perspectives for further investigation. Furthermore, our data have also shown the use of more than one plant part from the same plant species. For instance, *J*. *curcas* leaves and stem have been used to treat hyperuricemia and mouth ulcer, respectively.

**Fig 4:**
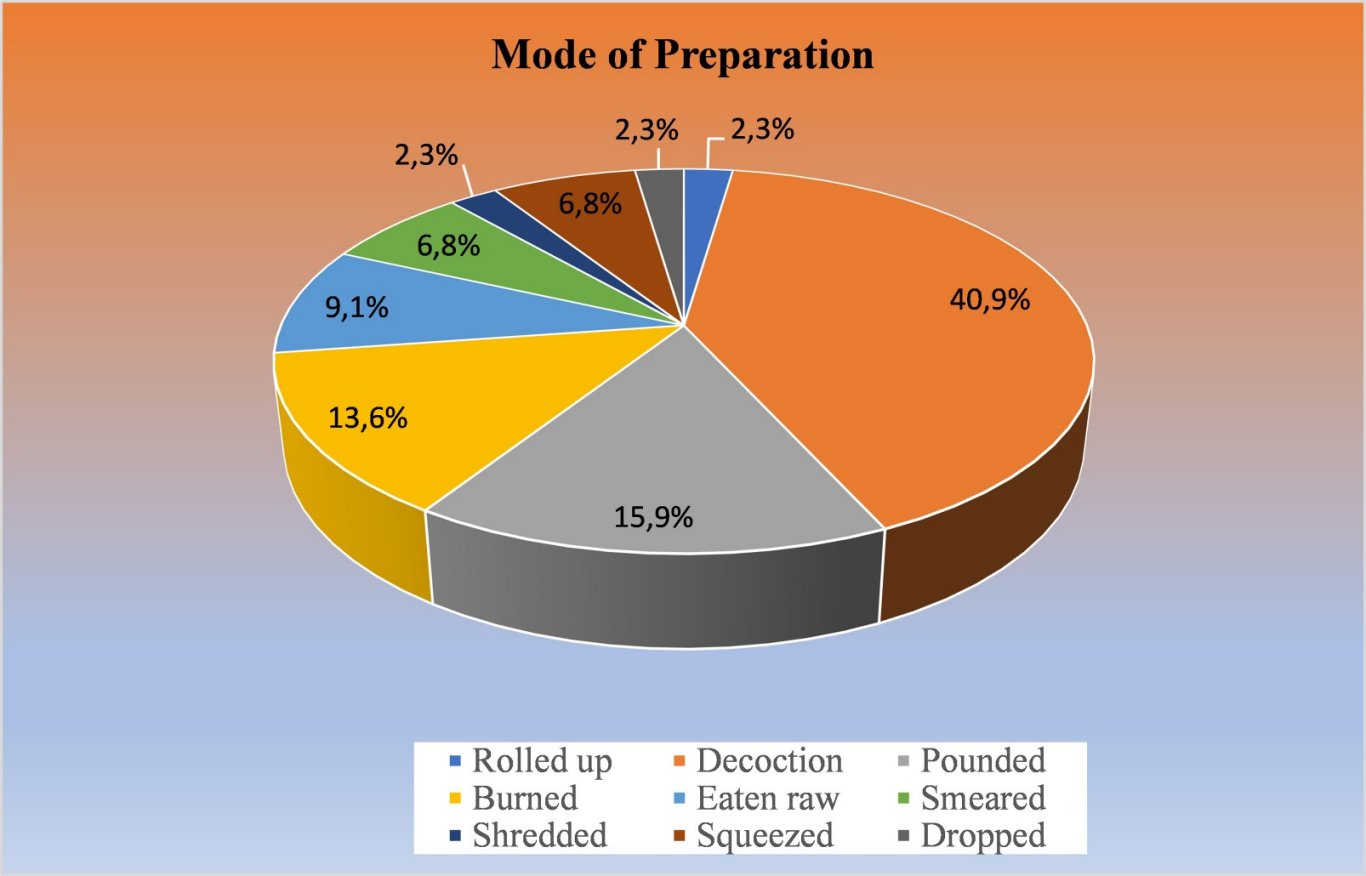
Mode of preparation of the medicinal plants used by the people of Tengger in Ngadisari village, Indonesia.

People from the Ngadisari village use many methods to prepare plant parts before using them as herbal medicine. The decoction is considered the main mode of preparation (40.9%), followed by pounding (15.9%) and burning (13.6%). Meanwhile, eating raw (9.1%) and smearing (6.8%) contribute and of the total mode of preparation in the present study. Other miscellaneous modes of preparations constitute the remaining 13.6% (Fig 4). Some other studies have also mentioned the same results, where the most common method of preparation is decoction [61–63]. Simple, easy handling and inexpensive are the major reasons why this mode of preparation is widely used by society [64]. Moreover, other reports also demonstrated that decoction might increase the efficiency of plant extraction and therefore increase its bioactivity [65].

Some plants can be prepared without any processing. For example, leaves of *A*. *graveolens* are eaten raw to reducing hypertension symptoms and leaves of *A*. *vulgaris* are applied directly by clogging into the nose to stop nosebleeds. All this local knowledge is preserved and applied by the people of Tengger in Ngadisari village. This practice is common in other regions in Indonesia such as in Madura and Bali [5, 66].

## Conclusions

Our results highlighted the use of medicinal plants by people from the Ngadisari village, Indonesia. A total of 30 medicinal plant species were recorded in the present study. They belong to 20 different families, where Poaceae and Zingiberaceae were the most representative families. A high number of plant species were used for treating internal medical diseases, respiratory-nose, ear, oral/dental, and throat problems. Leaves were the most popular plant part used and decoction was the most common method of preparation. These findings indicated potential roles of medicinal plants used in the Ngadisari village. Furthermore, our study characterized the cultural values of the people of the Ngadisari village. The species use, family use values and fidelity levels presented here may be used to further support plant conservation and pharmacological studies for new drug discovery. Out of all the plants we reported, approximately 26.7% were novel medicinal plants. In addition, 71.8% of the plant uses we documented of medicinal species were also novel. Some highly cited species recorded in our study warrant further biochemical analyses to evaluate their bioactive substances. Moreover, *in vitro* plant tissue culture could also be used as an alternative way to conserve medicinal plants documented in this study. Finally, the information we obtained could enable the local communities to develop, market, and profit from dried herbal products, which then substantially improving the collective revenue of the local society.
